# Sphingoid base in pineapple glucosylceramide suppresses experimental allergy by binding leukocyte mono‐immunoglobulin‐like receptor 3

**DOI:** 10.1002/jsfa.11610

**Published:** 2021-11-11

**Authors:** Ayumi Takemura, Nobuaki Ohto, Hiroshige Kuwahara, Masashi Mizuno

**Affiliations:** ^1^ Department of Agrobioscience, Graduate School of Agricultural Science Kobe University Kobe Japan; ^2^ Maruzen Pharmaceuticals Co. Ltd. Fukuyama Japan

**Keywords:** co‐culture system, glucosylceramide, leukocyte mono‐immunoglobulin‐like receptor 3, passive cutaneous anaphylaxis reaction, sphingoid base

## Abstract

**BACKGROUND:**

The increase in patients suffering from type I hypersensitivity, including hay fever and food allergy, is a serious public health issue around the world. Recent studies have focused on allergy prevention by food factors with fewer side effects. The purpose of this study was to evaluate the effect of dietary glucosylceramide from pineapples (P‐GlcCer) on type I hypersensitivity and elucidate mechanisms.

**RESULTS:**

Oral administration of P‐GlcCer inhibited ear edema in passive cutaneous anaphylaxis reaction. In a Caco‐2/RBL‐2H3 co‐culture system, P‐GlcCer inhibited *β*‐hexosaminidase release from RBL‐2H3 cells. The direct treatment of P‐GlcCer on RBL‐2H3 did not affect *β*‐hexosaminidase release, but sphingoid base moiety of P‐GlcCer did. These results predicted that sphingoid base, a metabolite of P‐GlcCer, through the intestine inhibited type I hypersensitivity by inhibiting mast cell degranulation. In addition, the inhibitory effects of P‐GlcCer on ear edema and degranulation of RBL‐2H3 cells were canceled by pretreatment of leukocyte mono‐immunoglobulin‐like receptor 3 (LMIR3)‐Fc, which can block LMIR3‐mediated inhibitory signals.

**CONCLUSION:**

It was demonstrated that a sphingoid base, one of the metabolites of P‐GlcCer, may inhibit mast cell degranulation by binding to LMIR3. The oral administration of P‐GlcCer is a novel and attractive food factor that acts directly on mast cells to suppress allergy. © 2021 The Authors. *Journal of The Science of Food and Agriculture* published by John Wiley & Sons Ltd on behalf of Society of Chemical Industry.

**ABBREVIATIONS**
4,8‐SD: 4,8‐sphingadienine
CMC: carboxymethyl cellulose
DNP‐IgE: anti‐dinitrophenyl IgE
LMIR3: leukocyte mono immunoglobulin like receptor 3
PCA: passive cutaneous anaphylaxis
P‐GlcCer: dietary glucosylceramide from pineapples
SB: Siraganian buffer
TGF‐β: transforming growth factor‐β
TNP‐IgE: 2, 4, 6‐trinitrophenyl‐IgE

## INTRODUCTION

Recently, the number of patients suffering allergic diseases has increased.^1^ Allergy, also called hypersensitivity, is defined as a disease following a response by the immune system to an otherwise innocuous antigen. Hypersensitivity reactions are classified into four types (I, II, III, and IV), which are different in terms of the disease manifestation and pathological processes.^2^, ^3^ Type I hypersensitivity, often called immediate‐type hypersensitivity, including hay fever, food allergy, and asthma, is characterized by immunoglobulin E (IgE)‐mediated reaction. The number of patients suffering from type I hypersensitivity diseases increases continuously,^4^ and type I hypersensitivity diseases are major health problems in the world. Efficient treatment of the most common type I hypersensitivity diseases is with the use of antihistamines and corticosteroids. However, sometimes they can cause unpleasant side effects, such as an increased appetite, mood changes, and difficulty sleeping,^5^ and rarely can induce hypersensitivity reactions.^6^, ^7^ Therefore, development of treatment methods with fewer side effects is desired, and many studies have focused on food factors.

Glucosylceramide (GlcCer) is a kind of glycosphingolipid that exists widely as a component of cell membranes.^8^ The chemical constituents of GlcCer include a glucose moiety bound to a ceramide skeleton, where fatty acid is combined to an amino group of a sphingoid base comprising a long‐chain alcohol.^9^ GlcCer is found in plants, animals, and fungi and is enriched in human epidermis as one of the major lipid components.^10^, ^11^ In particular, plants – including grains such as rice and corn, pulses such as soybeans, root vegetables such as konjac, and fruits such as apples – enable us to routinely take GlcCer from various ingredients. Recently, GlcCer from plants has been recognized as a functional food component. It has been reported that dietary GlcCer improves skin barrier function,^12^ has an anti‐inflammatory effect against atopic dermatitis^3^ and colitis,^14^ and suppresses messenger RNA expression of the proinflammatory cytokines interleukin‐1*β* and interleukin‐6.^15^

The immunoglobulin‐like receptors provide positive and negative regulation of immune cells upon recognition of various ligands.^16^, ^17^ The leukocyte mono‐immunoglobulin‐like receptor (LMIR) family belongs to the paired immune receptors.^18^ One of them, LMIR3, is mainly expressed on myeloid cells, such as granulocytes, dendritic cells, and mast cells.^18^, ^19^ LMIR3 delivers an inhibitory signal in a mast cell via its two immunoreceptor tyrosine‐based inhibitory motifs and a single immunoreceptor tyrosine‐based switch motif that can recruit Src homology 2 domain‐containing protein phosphatase‐1 and/or ‑2.^20^ Lipids have been suggested as LMIR3 ligands, and ceramide has recently been identified as a possible ligand.^21^, ^22^ In addition, it has been reported that the LMIR3 negatively regulates mast cell activation via an inhibitory signal.^23^ Medications targeting the inhibitory receptors, such as LMIR3, that inhibit mast cell activation may be an effective treatment for type I hypersensitivity.

In a previous study in our laboratory, it was shown that a metabolite from dietary GlcCer from pineapples (P‐GlcCer) improves skin barrier function.^24^ Furthermore, it has been revealed that P‐GlcCer stimulates the production of collagen in skin and improves skin barrier function.^25^ However, its effect on hypersensitivity reactions has not been reported. The aim of this study was to evaluate the effect of dietary P‐GlcCer on Type I hypersensitivity by focusing on the involvement of sphingoid base, a metabolite of P‐GlcCer, and LMIR3, and to elucidate the mechanism.

## MATERIALS AND METHODS

### Reagents

Dulbeccoʼs modified Eagleʼs medium (high glucose) with glutamine was purchased from Wako Pure Chemical Industries (Osaka, Japan). RPMI 1640 medium, minimal essential medium with non‐essential amino acids, and trypsin were purchased from Gibco BRL (Grand Island, NY, USA). Anti‐dinitrophenyl (DNP) IgE, *p‐*nitrophenyl *N*‐acetyl‐*β*‐d‐glucosaminide, and DNP–bovine serum albumin (BSA) were purchased from Sigma (St Louis, MO, USA). Fetal bovine serum was purchased from Biological Industries (Beit, Israel). 2,4,6‐Trinitrochlorobenzene was purchased from Tokyo Chemical Industry (Tokyo, Japan). Mouse anti‐2,4,6‐trinitrophenyl (TNP) IgE (clone: C38‐2) was purchased from BD Biosciences (Franklin Lakes, NJ, USA). LMIR3‐Fc (fragment, crystallizable) and Fc were purchased from R&D Systems (McKinley Place, MN, USA). Other chemicals and reagents were ordinary commercial and guaranteed products.

### Mice

BALB/c mice (4 weeks, female) were purchased from Japan SLC (Shizuoka, Japan). The mice were housed in an air‐conditioned animal room at 23 ± 2 °C and acclimated for 5 days before experiments. The mice were maintained in filter‐top cages in specific pathogen‐free conditions in Kobe University Life‐Science Laboratory with free access to laboratory chow (Rodent Diet CE‐2; CLEA Japan Inc., Tokyo, Japan) and water *ad libitum*. All animal experiments were approved and carried out in accordance with the Animal Experiment Ethnics Committee of Kobe University (registration number: 28‐11‐01).

### Passive cutaneous anaphylaxis reaction in mice

An IgE‐dependent passive cutaneous anaphylaxis (PCA) reaction was performed as described previously.^26^ Briefly, test samples were administered orally for 4 days. After 24 h, mice were sensitized by intravenous injection of anti‐TNP IgE antibody. After 30 min, the ear thickness was measured using a thickness micrometer (Peacock G‐1A; Ozaki Mfg. Co. Ltd, Tokyo, Japan) at baseline, and mice were challenged by painting of 1.6% (w/v) 2,4,6‐trinitrochlorobenzene in acetone–olive oil (1:1) as an antigen onto the surface of an earlobe. The ear thickness was measured again 2 h later. Ear edema was calculated according to differences in ear thickness before and after antigen challenge. To investigate the involvement of ceramide in the inhibitory effect of P‐GlcCer on the PCA reaction, each mouse was injected intravenously every day with LMIR3‐Fc or Fc (1 μg) 1 h after oral administration of P‐GlcCer and then injected with anti‐TNP IgE. P‐GlcCer (4 mg) was suspended in 0.5% carboxymethyl cellulose aqueous solution and administered to each mouse orally (150 μL day^−1^) using a gastric feeding tube. Mice to be used as a positive control were each orally administered luteolin (400 μg day^−1^) and F‐fucoidan (200 μg day^−1^).

### Measurement of transforming growth factor‐*β* in serum

Whole blood was left undisturbed for 30 min at 22–25 °C, maintained overnight at 4 °C, and subsequently centrifuged at 1200 × *g* at 4 °C for 15 min. Supernatants were collected as blood serum. Serum samples were stored at −80 °C until analysis. Serum transforming growth factor (TGF)‐*β* levels were measured using a0 Promega (Madison, WI, USA) enzyme‐linked immunosorbent assay kit in accordance with the manufacturerʼs standard protocol.

### *β*‐Hexosaminidase assay

To evaluate anti‐allergy effects of test samples, an *in vitro* assay using RBL‐2H3 cells was performed in accordance with previous study.^27^ RBL‐2H3 cells were seeded at 2.0 × 10^5^ cells per well onto 24‐well tissue culture plates in RPMI 1640 and incubated overnight. Then cells were washed three times with Siraganian buffer (SB; 119 mmol L^−1^ sodium chloride, 5 mmol L^−1^ potassium chloride, 0.4 mmol L^−1^ magnesium chloride, 1 mmol L^−1^ calcium chloride, 40 mmol L^−1^ sodium hydroxide (NaOH), 25 mmol L^−1^ piperazine‐*N*,*N*′‐bis(2‐ethanesulfonic acid), 5.6 mmol L^−1^ glucose, 0.1% BSA, pH 7.2) and exposed to test sample solutions for 2 h at 37 °C. Then, RBL‐2H3 cells were then incubated with 1 μg mL^−1^ at a final concentration of anti‐DNP IgE for 1 h. After replacing all media with SB, the cells were challenged with 500 μL per well of 100 ng mL^−1^ DNP–BSA for 1 h at 37 °C. The plate was cooled in an ice bath for 10 min to stop degranulation responses. The supernatant (50 μL) was transferred to a 96‐well plate and then incubated with an equal volume of substrate solution (2 mmol L^−1^ *p*‐nitrophenyl‐*N*‐acetyl‐*β*‐d‐glucosaminide in 0.2 M citrate buffer at pH 4.5) for 1 h at 37 °C. After adding 100 μL well of stop buffer (0.2 mol L^−1^ glycine–NaOH, pH 13.0), the absorbance at 405 nm was measured using a microplate reader. The percentage of *β*‐hexosaminidase released into the supernatants was calculated as a percentage of the degranulation group.

### Caco‐2/RBL‐2H3 cells co‐culture system

The co‐culture system composed of Caco‐2 and RBL‐2H3 cells was carried out based on our previous study.^27^ Caco‐2 cells were seeded at 0.7 × 10^5^ cells per well onto 24‐well Transwell insert plates (0.03 cm^2^, 0.4 μm pore size; Corning Costar Corp., Cambridge, MA, USA). Cell culture media were changed every 3 days until the transepithelial electrical resistance value of Caco‐2 cells reached 300 Ω cm^2^, which was measured using a Millicell‐ERS Voltohmmeter (Merck KGaA, Darmstadt, Germany). RBL‐2H3 cells were seeded at 2.0 × 10^5^ cells per well onto 24‐well tissue culture plates in RPMI 1640 and incubated overnight. Cells were then washed three times with SB, and Transwell inserts on which Caco‐2 cells had been cultured were added into the plate wells preloaded with RBL‐2H3 cells. Furthermore, 0.2 mL of RPMI 1640 or test sample solution was applied to the apical side. For assessing the influence of blocking on LMIR3‐mediated inhibitory signals, 5 μg mL^−1^ of LMIR‐Fc or Fc was simultaneously applied to the basolateral side. After incubation for 6 h, RBL‐2H3 cells were incubated with a 1 μg mL^−1^ final concentration of anti‐DNP IgE for 1 h. After replacing all media with SB, RBL‐2H3 cells were challenged with a 100 ng mL^−1^ final concentration of DNP–BSA for 1 h at 37 °C. The plate was cooled in an ice bath for 10 min to stop degranulation responses and then subjected to *β*‐hexosaminidase assay.

### Preparation of P‐GlcCer and synthesis of 4,8‐sphigadienine (4,8‐SD)

P‐GlcCer was prepared as described previously by Kuwata *et al*.^24^ The chemical structure of sphingolipid bases varies widely among species, and 4,8‐sphigadienine (4,8‐SD) (d18:2) is most abundant in plant species.^28^ (4*Z*,8*Z*)‐Sphinga‐4,8‐dienine (d18:2^4*Z*,8*Z*^) was synthesized according to the reported methods.^29^, ^30^

### Statistical analysis

Each result was expressed as the mean plus/minus the standard error. Statistical significance between any two groups was analyzed using Studentʼs *t*‐test. Statistical significance between more than two groups was analyzed by one‐way analysis of variance and the Tukey–Kramer test. Statistical significance was defined as *P* < 0.05.

## RESULTS

### The inhibitory effect of P‐GlcCer on type I hypersensitivity

**Figure 1:**
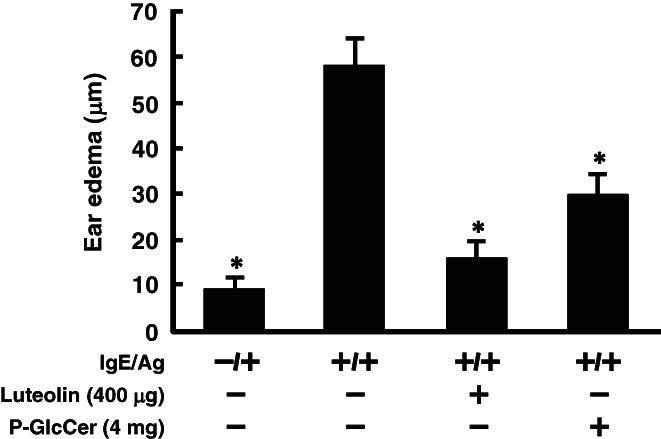
Effect of pineapple glucosylceramide (P‐GlcCer) on the passive cutaneous anaphylaxis reaction. P‐GlcCer (4 mg per mouse) and luteolin (400 μg per mouse) were orally administered to mice for 4 days before anti‐2,4,6‐trinitrophenyl immunoglobulin E (IgE) sensitization. After 30 min, mice were challenged by painting 1.6% (w/v) 2,4,6‐trinitrochlorobenzene (Ag) in acetone–olive oil (1:1) onto the surface of an earlobe. Ear edema was calculated according to differences in ear thickness before and after antigen challenge. Values represent means plus/minus standard error (*n* = 4 or 5). *P < 0.05 *versus* the degranulation group.

**Figure 2:**
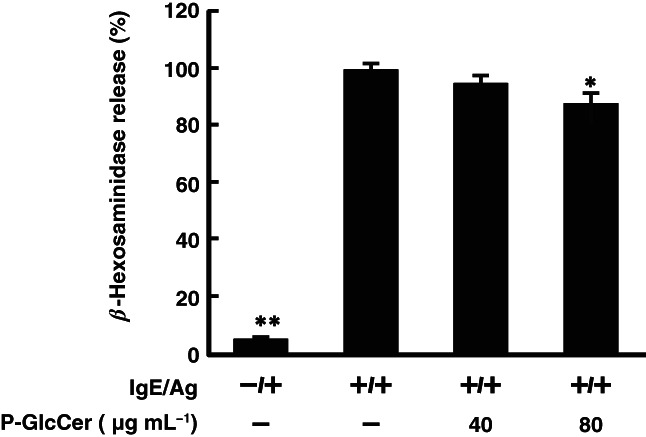
Effect of pineapple glucosylceramide (P‐GlcCer) on mast cell degranulation in a Caco‐2/RBL‐2H3 cells co‐culture system. P‐GlcCer (40, 80 μg mL^–1^) was applied to the apical side of the Caco‐2/RBL‐2H3 cells co‐culture system. After 6 h, Transwell inserts were removed and then RBL‐2H3 cells were sensitized with anti‐dinitrophenyl (DNP) immunoglobulin E (IgE) for 4 h. After all media were replaced with Siraganian buffer, degranulation of RBL‐2H3 cells was evoked by DNP–bovine serum albumin. Values represent means plus/minus standard error (*n* = 3). **P < 0.01, *P < 0.05 *versus* the degranulation group. Ag: 2,4,6‐trinitrochlorobenzene.

As the PCA reaction is a simple method to investigate the inhibitory effects of ingested test samples on type I hypersensitivity, the PCA reaction was performed in mice to evaluate inhibition effects of dietary P‐GlcCer in an *in vivo* experiment. Luteolin was used as a positive control, since it has been reported to inhibit an ear edema in the PCA reaction.^27^ Oral administration of P‐GlcCer for 4 days significantly inhibited ear edema compared with the degranulation group (Fig. 1). Furthermore, the Caco‐2/RBL‐2H3 co‐culture system was used in an *in vitro* experiment to investigate whether P‐GlcCer inhibits mast cell degranulation through intestinal epithelial cells. As shown in Fig. 2, P‐GlcCer (80 μg mL^−1^) significantly inhibited degranulation of RBL‐2H3 cells. These results indicated that P‐GlcCer inhibited hypersensitivity reaction by inhibiting mast cell degranulation.

### Possibility of TGF‐*β* production by P‐GlcCer on type I hypersensitivity

**Figure 3:**
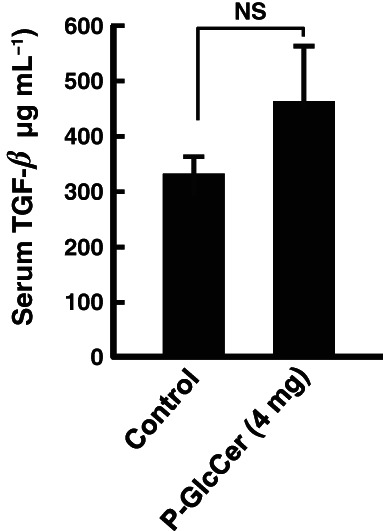
Effect of pineapple glucosylceramide (P‐GlcCer) on transforming growth factor (TGF)‐*β* levels in mice. P‐GlcCer (4 mg per mouse) was orally administered to mice for 4 days, and the passive cutaneous anaphylaxis reaction was performed as described in the Materials and Methods section. Serum TGF‐*β* level was measured using an enzyme‐linked immunosorbent assay. Values represent means plus/minus standard error (*n* = 5). NS: no significance.

A previous study showed that oral administration of P‐GlcCer for a long period restored the reduced serum TGF‐*β*1 levels.^25^ TGF‐*β* is known to promote keratinocyte migration, which is essential for the reconstruction of the cutaneous barrier after skin injury.^31^, ^32^ TGF‐*β* acts as a negative regulator of mast cell function, in part by decreasing Fc*ε*RI expression.^33^ During allergic reactions, much of mast cell activation is mediated through FcεRI, a receptor that binds to monomeric IgE on the mast cell surface.^34^ It was hypothesized that P‐GlcCer suppressed ear edema by TGF‐*β* production. To confirm this hypothesis, TGF‐*β* levels in serum were measured using an enzyme‐linked immunosorbent assay. Oral administration of P‐GlcCer for 4 days did not show significant changes in TGF‐*β* contents compared with the group with ear edema (Fig. 3). Thus, oral administration of P‐GlcCer in a short period did not affect TGF‐*β* contents in blood any differently than long‐term oral administration did.

### Inhibition of type I hypersensitivity by P‐GlcCer through LMIR3

**Figure 4:**
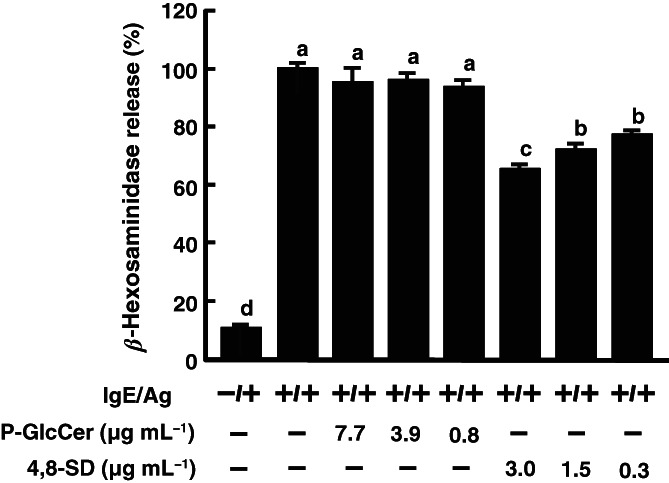
*β*‐Hexosaminidase release from RBL‐2H3 by treatment of pineapple glucosylceramide (P‐GlcCer) and 4,8‐sphingadienine (4,8‐SD). P‐GlcCer and 4,8‐SD were applied directly to RBL‐2H3 cells. After 2 h, RBL‐2H3 cells were sensitized with anti‐dinitrophenyl (DNP) immunoglobulin E (IgE) for 4 h. After all media were replaced with Siraganian buffer, degranulation of RBL‐2H3 cells was evoked by DNP–bovine serum albumin. Values represent means plus/minus standard error (*n* = 3). Items with different letters are significantly different (*P* < 0.05). Ag: 2,4,6‐trinitrochlorobenzene.

It is well known that GlcCer is enzymatically hydrolyzed to smaller components (such as ceramide, sphingoid base, and fatty acid) and, thereafter, these components are absorbed into the intestinal lumen.^35^ Therefore, it was predicted that P‐GlcCer itself or metabolites of P‐GlcCer acted to inhibit type I hypersensitivity. Moreover, it was indicated that 4,8‐SD, a metabolite of GlcCer, is the active compound mediating the improvement of skin barrier function.^24^ In order to confirm whether GlcCer or 4,8‐SD inhibits degranulation, each compound was directly pretreated with RBL cells, and then β‐hexosaminidase activity was measured. As shown in Fig. 4, 4,8‐SD suppressed *β*‐hexosaminidase release from RBL‐2H3 at a concentration of about 0.3 μg mL^−1^ (equivalent to 1 μmol L^−1^), whereas P‐GlcCer did not inhibit even at about 7.7 μg mL^−1^ (equivalent to 10 μmol L^−1^). These results suggested that 4,8‐SD may inhibit mast cell degranulation.

**Figure 5:**
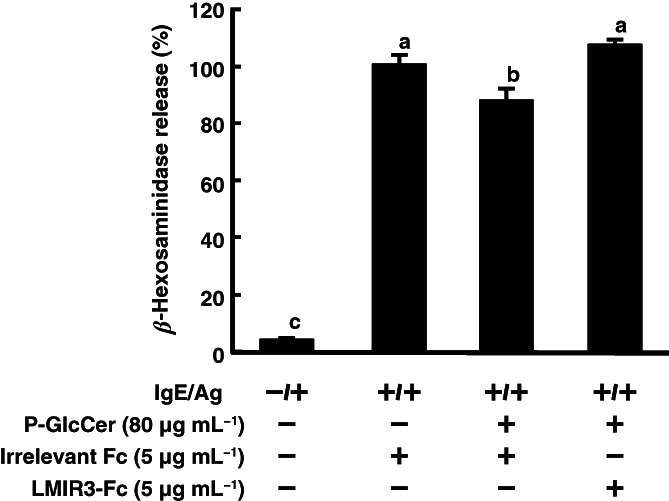
Influence of leukocyte mono‐immunoglobulin‐like receptor 3 (LMIR3)‐Fc treatment on *β*‐hexosaminidase in a Caco‐2/RBL‐2H3 cells co‐culture system. Pineapple glucosylceramide (P‐GlcCer; 80 μg mL^–1^) was applied to the apical side and 5 μg mL^−1^ of LMIR‐Fc or irrelevant‐Fc was applied to the basolateral side of the Caco‐2/RBL‐2H3 cells co‐culture system for 6 h. After Transwell inserts were removed, RBL‐2H3 cells were sensitized with anti‐dinitrophenyl (DNP) immunoglobulin E (IgE) for 4 h. Degranulation of RBL‐2H3 cells was evoked by DNP–bovine serum albumin. Values represent means plus/minus standard error (*n* = 3). Items with different letter are significantly different (*P* < 0.05). Ag: 2,4,6‐trinitrochlorobenzene.

**Figure 6:**
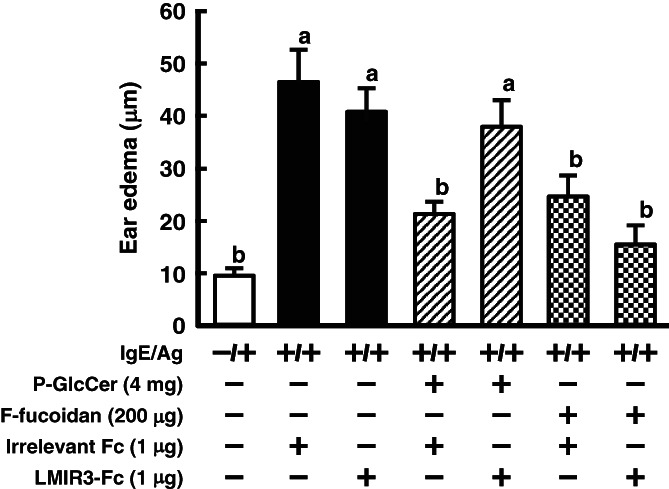
Influence of leukocyte mono‐immunoglobulin‐like receptor 3 (LMIR3)‐Fc on ear edema inhibition by pineapple glucosylceramide (P‐GlcCer) in the passive cutaneous anaphylaxis reaction. P‐GlcCer (4 mg per mouse) and F‐fucoidan (200 μg per mouse) were orally administered to mice for 4 days. Simultaneously, LMIR‐Fc or Fc (1 μg per mouse) was intravenously injected for 4 days at 1 h after oral administration of P‐GlcCer. At day 4, mice were sensitized with anti‐2,4,6‐trinitrophenyl immunoglobulin E (IgE) by intravenous injection. After 30 min, mice were challenged by painting 1.6% (w/v) 2,4,6‐trinitrochlorobenzene (Ag) in acetone–olive oil (1:1) onto the surface of an earlobe. Ear edema was calculated according to differences in ear thickness before and after antigen challenge. Values represent means plus/minus standard error (*n* = 4). Items with different letters are significantly different (*P*< 0.05).

Ceramide has been reported to be a ligand for LMIR3 on mast cells.^23^ This receptor is highly expressed on mast cells.^19^ Since 4,8‐SD is a component of ceramide, it was predicted that 4,8‐SD may bind LMIR3 to negatively regulate mast cell activation. To confirm this hypothesis, P‐GlcCer was added on the apical side of the Caco‐2/RBL‐2H3 cells co‐culture system, to which LMIR3‐Fc or irrelevant Fc was simultaneously added on the basolateral side prior to sensitizing with anti‐DNP IgE. As expected, the inhibition of *β*‐hexosaminidase release by P‐GlcCer was canceled by LMIR3‐Fc treatment (Fig. 5). Moreover, the PCA reaction was used to confirm the influence of LMIR3‐Fc on type I hypersensitivity. The inhibitory effect of P‐GlcCer on ear edema was canceled by intravenous injection of LMIR3‐Fc (Fig. 6). However, treatment with F‐fucoidan, which was known to suppress mast cell activation by interacting with glectin‐9 but not LMIR3, maintained the inhibition of ear edema by P‐GlcCer.^26^ It was shown that suppression of ear edema by LMIR3‐Fc is specific to P‐GlcCer. These results suggested that 4,8‐SD, one of the metabolites of P‐GlcCer, may inhibit mast cell degranulation via an LMIR3‐mediated inhibitory signal.

## DISCUSSION

Type I hypersensitivity diseases, including hay fever, food allergy, and asthma, are major health problems around the world, and the number of patients suffering from these diseases increases continuously.^4^ Against this background, effective treatments for these diseases have been sought, and in recent years, many studies have focused on food factors. GlcCer from plants has attracted intense interest as a functional food component having an improvement effect on skin barrier^25^ and anti‐inflammatory effect,^13^ but the effect on type I hypersensitivity has not been investigated.

In this study, it was shown that oral administration of P‐GlcCer for 4 days had an inhibitory effect on ear edema in the PCA reaction (Fig. 1). In addition, the Caco‐2/RBL‐2H3 cells co‐culture system showed that P‐GlcCer (80 μg mL^−1^) had an inhibitory effect on degranulation of RBL‐2H3 cells (Fig. 2). However, direct treatment of P‐GlcCer on RBL‐2H3 did not show any activity (Fig. 4). These findings suggested that metabolites of P‐GlcCer through the intestine had an influence on mast cell degranulation. Indeed, Kuwata *et al*.^24^ showed that 4,8‐SD, an intestinal metabolite of P‐GlcCer, ameliorated dry skin symptoms. Moreover, we confirmed that long‐term oral administration of P‐GlcCer, such as 4 weeks, has been associated with improved epidermal barrier function by restoring depressed serum TGF‐*β* to normal levels.^25^ Gomez *et al*.^33^ reported that TGF‐*β*1 acts as a negative regulator of mast cell function by decreasing FcεRI expression which had high affinity to IgE. Because TGF‐β secreted by oral administration of P‐GlcCer was assumed to play the role in prevention of PCA reaction, TGF‐β content was measured and no significant changes were observed after short period of P‐GlcCer administration of 4 days (Fig. 3). Thus, TGF‐β was found not to be a candidate to ameliorate PCA reaction.

In this study, 4,8‐SD treatment applied directly to RBL‐2H3 had an inhibitory activity on *β*‐hexosaminidase release, but P‐GlcCer did not (Fig. 4), suggesting that 4,8‐SD could be a candidate that can ameliorate allergy. GluCer derived from plants is enzymatically hydrolyzed by brush border enzymes in the gut lumen into metabolites such as ceramides and sphingoid bases.^35^ Therefore, it was assumed that the administered GlcCer is absorbed from the intestinal tract and metabolized to produce 4,8‐SD. It was reported that ceramide is a ligand for LMIR3, which negatively regulates mast cell activation via an inhibitory signal.^23^ Since 4,8‐SD is a component of ceramide, it was possible that 4,8‐SD is involved in LMIR3‐mediated suppression of mast cell degranulation. In the Caco‐2/RBL‐2H3 co‐culture system, the inhibitory effect of P‐GlcCer on degranulation of RBL‐2H3 cells was canceled by addition of LMIR3‐Fc on the basolateral side (Fig. 5). In the PCA reaction, the inhibitory effect of P‐GlcCer on ear edema was canceled by intravenous injection of LMIR3‐Fc (Fig. 6). In contrast, oral administration of F‐fucoidan, which inhibited ear edema through galectin‐9 secretion into blood,^26^ showed no changes with or without LMIR3‐Fc treatment, indicating that LMIR3 contributed to the anti‐allergic activity of P‐GlcCer. Although we have not been able to ascertain in this study whether 4,8‐SD is directly involved in LMIR3 signaling, it was suggested that sphingoid base, one of the metabolites of P‐GlcCer, may inhibit mast cell degranulation via an LMIR3‐mediated inhibitory signal, thereby ameliorating the PCA reaction.

## CONCLUSIONS

Research into the inhibition of allergy by food factors has often been focused on restoring the Th1/Th2 balance disrupted by allergens. However, P‐GlcCer is a novel and attractive food factor that acts directly on mast cells to suppress allergy. We conclude that P‐GlcCer may be an effective treatment of type I hypersensitivity.

## FUNDING

This research did not receive any specific grant from funding agencies in the public, commercial, or not‐for‐profit sectors.

## CONFLICT OF INTEREST

The authors declare that they have no known competing financial interests or personal relationships that could have appeared to influence the work reported in this paper.

## AUTHOR CONTRIBUTIONS

MM conceived and designed the experiments. AT and NO performed the experiments. AT analyzed the data. NO and HK contributed reagents/materials/analysis tools. AT and MM wrote the paper.
